# Respiratory Syncytial Virus: The Influence of Serotype and Genotype Variability on Clinical Course of Infection

**DOI:** 10.3390/ijms18081717

**Published:** 2017-08-06

**Authors:** Silvia Vandini, Carlotta Biagi, Marcello Lanari

**Affiliations:** 1 Pediatrics and Neonatology Unit, Imola Hospital, 40026 Imola, Italy; s.vandini@ausl.imola.bo.it; 2 Department of Pediatric Emergency, S. Orsola-Malpighi Hospital, University of Bologna, 40138 Bologna, Italy; marcello.lanari@unibo.it

**Keywords:** respiratory syncytial virus, genotype, children, lower respiratory tract infections, bronchiolitis, monoclonal antibodies, vaccines

## Abstract

Respiratory syncytial virus (RSV) belongs to the recently defined *Pneumoviridae* family, Orthopneumovirus genus. It is the leading cause of acute bronchiolitis and one of the most common causes of infant viral death worldwide, with infection typically occurring as recurrent seasonal epidemics. There are two major RSV subtypes, A and B, and multiple genotypes, which can coexist during RSV epidemic season every year and result in different disease severity. Recently, new RSV genomic sequences and analysis of RSV genotypes have provided important data for understanding RSV pathogenesis. Novel RSV strains do spread rapidly and widely, and a knowledge of viral strain-specific phenotypes may be important in order to include the more virulent strains in future therapeutical options and vaccine development. Here we summarize recent literature exploring genetic and molecular aspects related to RSV infection, their impact on the clinical course of the disease and their potential utility in the development of safe and effective preventive and therapeutic strategies.

## 1. Introduction

Respiratory syncytial virus (RSV) is a highly contagious RNA virus that represents the main cause of respiratory tract infection in children worldwide. A review by Nair et al. [1] estimated that in 2005, 33.8 million acute RSV infections occurred worldwide in infants younger than five years, of whom 66,000–199,000 died (99% in the developing countries). Most infants at 18 months of age (87%), and virtually all children reaching three years of age have already developed specific anti-RSV antibodies [1]. A recent study by Anderson et al. [2] reported a hospitalization rate close to 3.4% for infants born at 33–35 weeks of gestational age; severe cases required ICU admission (22%) and mechanical ventilation (12%). RSV manifestations range from coryza to severe lower respiratory tract infection (LRTI) including bronchiolitis and pneumonia, which can lead to hospitalization and respiratory failure [1,3]. Moreover, infant hospitalizations due to RSV infection have been associated with chronic wheeze and asthma disease as well as with a reduction in respiratory function in school-aged children [2,4]. RSV infections are particularly severe in high risk pediatric populations (infants affected by chronic lung disease, congenital heart disease, neuromuscular disorders, primary and acquired immunodeficiencies, and infants born prematurely) [5,6,7]. High risk patients often require ICU admission and respiratory support. For these reasons, they are candidates to receive immunoprophylactic therapy with monoclonal antibodies (Palivizumab) according to updated international guidelines [8,9]. Nevertheless, it has been shown that the majority of hospitalized RSV infected children do not fully meet the criteria for immunoprophylaxis, and to date there are no licensed vaccines available. Furthermore, none of the proposed etiologic treatments has been demonstrated to be truly effective, thereby reducing therapeutical options of acute RSV infections to supportive therapy (mainly oxygen supplementation and hydration). No antiviral agents are routinely used [10]. Ribavirin is up to now the only antiviral drug approved for the treatment of severe RSV infection, but its use is limited because of its side effects and teratogenic potential in occupationally exposed workers [11]. Thus, vaccines and etiologic therapies are needed to decrease the RSV disease burden.

RSV is ubiquitous, with relatively uniform distribution worldwide. In temperate climates (i.e., many areas of the USA and Europe) it is responsible for annual outbreaks during in the late fall, winter and early spring, while in cold climates it is nearly continuous throughout the year [12]. Two antigenically different subtypes exist, A and B, and multiple genotypes, which can co-circulate during the same epidemic season. The clinical impact of viral factors during RSV infection is still controversial, as some studies have reported that different subtypes and genotypes lead to different disease severity [13,14,15,16], while others have shown that they have equivalent severity [17,18,19]. Finding a relationship between viral factors and clinical presentation may be important in order to include the more virulent strains in future vaccines and treatments.

Here, we discuss the characteristics of RSV focusing on the genetic and molecular aspects related to the infection, their impact on the clinical course of the disease as well as their potential utility in the development of safe and effective preventive and therapeutic strategies.

## 2. Characteristics of the Virus

RSV is an enveloped non-segmented negative-sense single-stranded RNA virus, and a member of the Orthopneumovirus genus and *Pneumoviridae* family.

The RNA of RSV contains 10 genes encoding 11 proteins. The envelope of the virus is formed by four proteins associated with the lipid bilayer: the matrix (M) protein, the small hydrophobic (SH) protein, and the two glycosylated surface proteins: the fusion (F) and the attachment glycoprotein (G). F and G proteins are crucial for virus infectivity and pathogenesis since the G protein is responsible for the attachment of the virus to respiratory epithelial cells, while the F protein determines the entry of the virus, by fusing viral and cellular membranes, as well as the subsequent insertion of the viral RNA into the host cell inducing the formation of the characteristic syncytia. Moreover, the F and G proteins stimulate the neutralizing antibody immune response by the host.

The G protein is a type II glycoprotein synthesized as a polypeptide composed by 300 amino acids (depending on the viral strain) with a single C-terminal hydrophobic domain and a large number of glycan added [20]. Three types of epitopes have been identified in the G protein by murine monoclonal antibodies: (I) conserved epitopes, detectable in all viral strains; (II) group-specific epitopes, expressed only by to the same antigenic group and (III) strain-specific epitopes, that are present only in specific strains of the same antigenic group and expressed in the C-terminal hypervariable region of the G protein ectodomain [21].

The F protein is a type I glycoprotein which has a structure comparable to the F proteins of other *Pneumoviridae* (e.g., metapneumovirus) and *Paramyxoviridae* (e.g., parainfluenza virus type 5) viruses. The F glycoprotein derives from an inactive precursor containing three hydrophobic peptides: (I) the N-terminal signal peptide, which mediates translocation of the nascent polypeptide into the lumen of the endoplasmic reticulum; (II) the transmembrane region near the C-terminus, which links F protein to the cell and viral membranes; and (III) the so-called fusion peptide, which inserts into the target cell membrane and determines the fusion process. The binding of prefusion F protein to the cell surface is followed by its activation and conformational changes, which leads to the fusion of the virion membrane with the host cell membrane.

There are two major RSV groups, A and B, which generally coexist early during an RSV epidemic season, even if temporal and geographic clustering may occur [22,23]. The antigenic variability between the two groups is determined by variations in the G glycoprotein (35% homology between G glycoprotein of strains A and B) [24]. For this reason, many antibodies targeted to G protein may be subtype specific, while antibodies against the F protein are cross-reactive for RSV A and B. RSV infections with group A are more frequent than those of RSV B and their transmissibility seems to be higher [25]. The existence of two groups, A and B, and their alternating infection incidences may play a role in the ability of RSV to infect previously exposed individuals and bypass preexisting immune responses [26,27]. Moreover, additional antigenic variability occurs within the two groups and many genotypes from each group have been described. To date, nucleotide sequence analysis of the G protein has led to the identification of 11 RSV-A (GA1-GA7, NA1, NA2, SAA1 and ON1) [23,28] and 23 RSV-B genotypes (GB1-GB4, SAB1-SAB4, URU1, URU2, BA1-BA12 and THB) [29,30,31,32]. Different genotypes can co-circulate during an epidemic season, and the predominance of one over the other varies by years and location [33,34]. In particular, the rapid spread of a novel RSV-A genotype (A/ON1, replacing the ancestor A/NA1) has recently been documented in a number of countries [32,35,36]. The rapid spread of the genotype ON1 may be related to the duplicated sequence within the G gene [36]. Agoti and colleagues [37] recently performed a phylogenetic analysis of the RSV genomes in children hospitalized for RSV infections in Kilifi, Kenya, using a novel full-genome deep-sequencing process. They found out that most RSV A variants were observed worldwide, while the local RSV B genomes encoded a high degree of variation that was not described in other parts of the world. Ongoing surveillance of the molecular epidemiology of RSV genotypes is important for detecting the emergence of new viral strains, predicting their clinical impact and selecting candidate strains for vaccine and therapeutics development.

## 3. Genetic Diversity and Clinical Impact

The clinical impact of viral factors during RSV infection is still controversial, as there are conflicting reports regarding the associations of different groups and genotypes with severity of infection.

Some studies have reported that RSV-A is associated with more severe clinical disease [13,38,39,40,41,42]. McConnochie et al. [39] observed that a higher percentage of patients with RSV-A infection required mechanical ventilation compared to those infected by RSV-B (12% vs. 1.6%, *p* = 0.01). Another 15 year study of 1209 hospitalized and ambulatory children with RSV infection revealed that children with group A infections required intensive care significantly more often than those infected by other RSV strains (15.4% vs. 8.3%, *p* = 0.008) [40]. Walsh and colleagues assessed the relationship between RSV groups and disease severity in 265 hospitalized infants over a 3-year period (1988–1991) and found that among infants without underlying medical conditions, those infected by RSV-A were at higher risk of requiring ventilation support and had greater disease severity than those infected by group B RSV [38]. Similarly, Papadopoulos et al. demonstrated a higher (more severe) clinical score index in RSV-A infection [41], on the basis of respiratory rate, wheezing, heart rate, difficulty in feeding and oxygen saturation. However, no statistically significant difference was found in duration of hospitalization or need of intensive care. A recent prospective, multicenter U.S. study of 1589 children hospitalized with bronchiolitis from 2007 through 2010 found no difference in disease severity between infants with RSV-A and RSV-B [13]. However, when the authors restricted the analysis to children with no co-infecting virus, they found that RSV-A was associated with a higher risk of intensive care treatment compared with subtype B. No differences were found between the subtypes regarding the hospital length-of-stay [13].

In 2013, data from the largest epidemiologic study conducted in 27 US emergency departments, showed that RSV-A positive patients were more likely to be admitted to the hospital or intensive care unit (47.7%) versus RSV-B (35.8%; *p* < 0.001), supporting suggestions from smaller studies that RSV-A may be more virulent than RSV-B. However, more quantitative assessments of disease severity are needed to determine whether RSV subtype is associated with increased disease severity [42].

On the contrary, other authors have reported that RSV-B infection is more severe [43]. Hornsleth et al. prospectively studied 105 children admitted to a pediatric department in Copenhagen due to RSV respiratory infections during three winter seasons, 1993–1995 [43]. RSV strains were typed and genotyped, respectively, by PCR and nucleic acid restriction analysis. They found an age-related difference in the disease severity: RSV B infections were associated with longer hospital stays, use of respiratory support and the presence of an infiltrate on a chest radiograph in infants zero to five months old. In a Vietnamese study [44], enrolling 235 cases with RSV-A and 13 cases with RSV B infection, subgroup B infection determined low hospitalization rate and lower clinical severity score than subgroup A infection in children. However, this study by Tran and colleagues analyzed data collected in a single epidemic season, so the results could be confounded by the predominant subgroups shift from year to year that may affect the immunity acquired against the previously circulating viruses.

Finally, other studies have shown that RSV-A and B infections have equivalent severity [18,19]. In a 3-year prospective study of 444 children with RSV bronchiolitis (Group A, 337; Group B, 107), no difference in severity between Group A and Group B infection was reported [19]. According to the results from 81 previously healthy infants hospitalized for RSV bronchiolitis, Fodha et al. concluded that disease severity was not associated significantly with RSV subgroup. Instead, it was likely to be determined by an interplay between host and virus factors, including RSV load [18].

A recent study suggested specific clinical presentations for the different RSV subtypes [45]. In studying 729 RSV-positive children (< 14 years old) hospitalized with respiratory illness in China, over a 3-year period, Liu et al. showed specific clinical presentations for RSV-A and RSV-B infections: bronchiolitis, dyspnea, coryza and gastrointestinal symptoms were found more frequently in the RSV-A positive patients, while the systemic influenza-like symptoms of chills, myalgia, rash, debility and headache were mainly associated with RSV-B.

Several recent studies have molecularly characterized circulating RSV genotypes in different cohorts. Genotyping of RSV-A and RSV-B viruses is based on the sequence variability of the gene encoding the G protein.

A recent study [46] of Pakistani children reported 71 subgroup A and 4 subgroup B strains. Strains were further identified as NA1/GA2 and BA, respectively. The analogies of nucleotides and aminoacids were relatively high among these strains (>90%). Both RSV-A and RSV-B isolates contained two potential N-glycosylation sites in the HVR2 domain of G protein. More recently, Eshagi et al. [47] explored the genetic variability of RSV-A circulating in Ontario during the 2010–2011 winter season, finding a novel genotype called ON1, consisting of a 72 nucleotide duplication in the C-terminal region of the attachment (G) glycoprotein. Another recent retrospective study [36] analyzed a German cohort of children finding that the majority of RSV-A strains (65%) belonged to the novel ON1 genotype.

Regarding the relationship between virulence and RSV strains, recent evidence shows that specific genotypes of subtype A are related to increased illness severity [14,15,16]. Martinello and colleagues collected data from 107 children aged 0–2 years with RSV disease (64 RSV-A and 43 RSV-B) enrolled during two winter seasons [15]. They found that the severity of illness did not differ between the two RSV subgroups (*p* = 0.086), but that the GA3 clade was associated with significantly greater severity of illness, compared with clades GA2 (*p* = 0.018) and subgroup B (*p* = 0.032). Another recent study [48] reported that the NA1 RSV genotype is associated with a higher hospitalization rate, more severe clinical course and higher viral load.

Esposito et al. investigated the virulence of the RSV A/ON1, a novel RSV-A genotype that has recently emerged in a number of countries [32,35,36]. They observed that children with RSV-A/ON infection presented less frequently with lower respiratory tract infection symptoms and were less frequently hospitalized compared with children with A/NA1. These data are supported by other studies [36,49,50], suggesting that RSV-A/ON1 is significantly less virulent than genotype A/NA1 [14]. Moreover, the genotype A/ON1 was significantly less virulent than the simultaneously circulating B/BA9 and B/BA10 viruses. This is in line with the findings of Panayiotou et al, who showed that children with RSV-A/ON1 infection had a lower severity score than patients with an RSV-B/BA infection [49].

However, the evidence regarding the severity of RSV-A/ON1 has been controversial. Yoshihara and colleagues [15] investigated the clinical impact of ON1 among hospitalized pediatric patients in Central Vietnam from January 2010 to December 2012. They compared the clinical characteristics of RSV-A/ON1 with NA1 genotype (123 vs. 138 cases). The length-of-stay was similar between the two groups, while ON1 cases were associated with greater risk of hospitalization, LRTI and radiologically confirmed pneumonia compared to the previously predominant NA1 genotype. The very different findings concerning the clinical impact of the emerging ON1 genotype may be a consequence of the recent RSV experience of each community or of the age range of the cohort.

The herd immunity may partially explain the controversial data reported in literature about the clinical impact of RSV groups and genotypes. Different subtypes and virus strains have been detected during local-community RSV epidemic season, and the dominant ones subsequently decrease in epidemics of the following years [25,26,27,37]. This pattern of dominance and replacement of virus strains in the same region may be explained by strain-specific herd immunity. Recent RSV experience of a community may have provided better immunity to one RSV subtype, or to one strain than another, with subsequent reduction in disease severity. Moreover, the conflicting reports regarding the associations of RSV group and genotype with clinical outcome may also depend on the age range of the cohort studies. Older children were more likely to experience a second or third infection rather than a primary infection, and they would be less likely to display severe disease [1,3]. The variations in viral proteins other than G and F may also contribute to pathogenicity and severity of illness, but their real importance is still unclear. Finally, additional investigations are needed to evaluate whether aminoacid changes in RSV proteins may help the virus to alter the antigenic characteristics leading to a different degree of virulence.

The ongoing Infant Susceptibility to Pulmonary Infections and Asthma Following RSV Exposure (INSPIRE) study is currently examining human and viral characteristics correlated with wheezing or asthma after infant RSV infection, with the aim to confirm the hypothesis that specific RSV strains may be related with different clinical course and long term sequelae as recurrent wheezing and early childhood asthma [51].

We summarized the correlation between clinical features and subtypes in Table 1, and between clinical features and genotypes in Table 2.

## 4. State of the Art for Active and Passive Prophylaxis against RSV

### 4.1. Monoclonal Antibodies

The observation that neutralizing antibodies against RSV in immune serum was effective in preventing severe RSV infections started from the development of RespiGam, a polyclonal RSV hyperimmune globulin (RSV-IGIV) derived from pooled human plasma containing high concentration of protective antibodies against RSV [52] The success of RSV-IGIV and the Food and Drug Administration approval in 1996, promoted the development of specific monoclonal antibodies (mAbs). Both RSV surface glycoproteins, G and F proteins, could be targets for neutralizing mAbs. However, because of its heterogeneity, G protein was observed to be not an effective candidate for passive immunization against RSV. In contrast to G protein, F protein is well conserved in different virus strains, and it is conserved over time and geographical regions [53]. Therefore, two different IgG1 mAbs neutralizing RSV F proteins have been developed: SB 209763 and Palivizumab.SB 209763 is an IgG1 neutralizing the C epitope. Its efficacy was studied in a large, multicenter, placebo-controlled clinical trial [54] on more than 800 American and European children that received a monthly dose 10 mg/kg. However, the study did not demonstrated a significant reduction in RSV-related hospitalizations, perhaps due to insufficient dose. Indeed, the trial by Meissner et al. [52] showed that the dose studied in the trial did not determine the achievement of a protective antibody titer and subsequently it was not effective to prevent RSV lower respiratory tract infection. The research on this antibody was not continued and currently palivizumab is the only mAb used for the prophylaxis of RSV infections.

Palivizumab is also an IgG1 monoclonal antibody directed against the RSV F protein, but to a different conserved epitope (A) [55,56]. Currently it represents the only monoclonal antibody approved for the prophylaxis of RSV infection. Palivizumab was reported to reduce by 55% RSV related hospitalization in preterm infants with chronic lung disease [57] and in infants with hemodynamically significant congenital heart disease [58]. Palivizumab prophylaxis is effective, but it is highly expensive; for this reason its use for prevention of severe RSV infections in high risk pediatric populations is limited to the developed countries, where its use is regulated by guidelines in several countries (i.e., USA, UK, Canada, Italy, Scotland, Spain, Japan, Argentina) [8,59,60,61,62,63,64].

More recent studies [65,66] reported that the more potent neutralizing sites are in the prefusion form of the F protein; for this reason, the determination of the prefusion RSV F structure and identifying the detection of neutralizing antibodies binding prefusion-specific antigenic sites became one of the most important objective for developing active and passive immunization strategies against RSV.

This result was also confirmed by the detection of antibodies from immortalized human T lymphocytes [67] specific for prefusion F that had higher neutralizing potency [64] than those obtained from both prefusion and postfusion F [68].

These recent findings lead to the development of a new monoclonal antibody, MEDI8897 [69]. MEDI8897 is a recombinant human IgG1 kappa monoclonal antibody binding the prefusion form of the RSV F protein [65] and is currently under clinical development for the prevention of RSV infections. MEDI8897 is derived from D25, another human monoclonal antibody that was reported to have approximately 100-fold greater potency than palivizumab in vitro [67].

### 4.2. Vaccines

Currently, no vaccines against RSV are available, but several candidates are in preclinical and clinical studies. Multiple observations support the feasibility of the RSV vaccine [70]: RSV infection is very frequent during the first year of life; infections recur throughout life but they have a less severe clinical course in older children and adults that can be explained by the increase of natural immunity; moreover, a reduced incidence of RSV during the neonatal period and the first months of life is related to higher levels of RSV-specific maternal antibody.

RSV vaccine development started in 1960 with an unsuccessful formalin-inactivateed vaccine [71] that induced a severe and, in two cases, lethal lung inflammatory response. However, a growing number of vaccines have been studied in the last decades with promising results [72,73,74].

Currently, several vaccine candidates are in preclinical studies, including live-attenuated and live-vectored vaccines, protein based vaccines (including whole-inactivated virus, subunit antigens that associate to form aggregate particles, and non-particle based subunit antigens), nucleic acid and gene-based vectors [75]. A promising strategy could be the immunization of pregnant women, as antibodies are transferred efficiently through the placenta. The aim is to increase the antibody protective titer in the mother that will protect the newborn during the first months of life. In this regard, a recent trial documented the safety and immunogenicity of a RSV recombinant F protein nanoparticle in women of childbearing age [76,77,78,79,80]. It was reported by several studies that the level of serum maternal antibodies against RSV is inversely related to the incidence of RSV infections and RSV-related hospitalization in infants during the first months of life [81,82,83,84].

The F protein is the major vaccine target for several reasons: (I) the strict requirement for F mediating virus entry; (II) the relative genetic and antigenic stability of F; and (III) the established efficacy of the anti-F mAb, Palivizumab, in the decrease of severe RSV disease in high-risk infants.

RSV F protein anchors the viral and host cell membranes through passing from the labile prefusion to the stable postfusion conformation. Structures of these conformations have been solved for RSV F protein [65,66,77,85]. In vitro studies reported that prefusion conformation determine a higher immune response [68], but it is highly instable and rapidly switches to the stable postfusion conformation [65,86]. The stabilization of the RSV F protein has recently been reached with some modifications that led to the formation of a stable prefusion molecule with modest expression levels, but a satisfactory immunogenicity in animal models [65]. A recent study [87] identified novel mutations thanks to the comprehension of the molecular basis of the prefusion instability. They developed a model with a few amino acid substitutions that increased the prefusion F protein stability and maintained the prefusion conformation. The immunogenicity of the stabilized F protein was then tested in mice obtaining an antibody titer 10-fold higher than the titer obtained with postfusion F protein.

An important concern in the development of RSV vaccine is the safety of the vaccine, and the absence of enhanced disease risk. A recent study [88] evaluated the response to RSV post-F and pre-F in combination with glucopyranosyl lipid A (GLA) integrated into stable emulsion (SE) (GLA-SE) and alum adjuvants in the cotton rat model. Immunization with optimal doses of RSV F antigens in these adjuvants determined the synthesis of high titers of neutralizing antibodies and complete lung protection without signs of alveolitis. Subsequently, an antigen dose de-escalation study was performed in the presence of both adjuvants. At low RSV F protein doses, alveolitis was unexpectedly observed within both cases. This occurred despite a protective antibody titer and without virus replication in the lungs. These data underline the need to investigate a pediatric RSV vaccine candidate safe from the risk of vaccine induced severe pulmonary disease, even in the presence of strong neutralizing activity.

Other vaccine development strategies include a chimeric Human Parainfluenza Virus 3 (HPIV3) vector expressing RSV F protein as a bivalent RSV/HPIV3 vaccine and demonstrating an improvement of pre-F stabilization, strategic manipulation of codon usage and efficient pre-F packaging into vector virions [89].

Another study [74] analyzed safety and immunogenicity of a vaccine against RSV vectored by chimpanzee adenovirus and modified vaccinia virus Ankara with encouraging results.

A vaccine candidate vectored by human adenovirus serotypes 26 and 35 encoding the F protein gene was developed by Janssen Pharmaceutical Companies [90,91]. The trials showed a strong cellular and humoral immune response without significant lung damage in cotton rats [90] and in humans in a very recent report from the pharmaceutical trials presented by Janssen Pharmaceutical Companies to the Vaccine and Related Biological Product Advisory Committee of the Food and Drug Administration [91].

Another promising vaccine candidate was developed by Hwang et al. [92]. They demonstrated the immunogenicity, efficacy, and safety of virus-like particle vaccines containing RSV F G or F and G proteins in cotton rats. RSV specific antibodies were effectively induced by the vaccine candidates, without pulmonary inflammation in rats immunized with FG and F vaccines, while G vaccine induced moderate alveolar inflammation with eosinophilia and mucus production.

Moreover, live-attenuated and live-vectored RSV vaccines are currently in clinical development (Dr. Peter Collins, NIH). Live vaccines are effective because they induce broad humoral and cellular immunity; they do not require adjuvants and are not reported to cause FI-RSV enhanced disease, because they express viral surface glycoproteins in their native conformations [93].

## 5. Future Directions

The clinical impact of RSV disease may depend on the incidence of infections due to the most virulent genotype during the epidemic season. The evaluation of RSV group is not enough to predict the risk of severe infection during epidemics, and specific studies planned to assess the virulence of the different genotypes are needed.

Future studies should focus on the analysis of RSV molecular epidemiology, evolution and transmission with the aim of defining the circulating viruses and characterizing the antigenic variation. These studies might provide important implications for vaccine development and for finding new strategies to control the burden of RSV disease and identifying other viral proteins that could be targets of neutralizing antibodies.

## Figures and Tables

**Table 1:** Principal studies evaluating the correlation between respiratory syncytial virus groups and severity of illness in children.

Study	Period of Study	Country	Study Population	Number of Cases	Results (*p* Value)
McConnochie et al. [39]	2 winter seasons (1985–1987)	Rochester, New York, United States	Infants hospitalized for acute respiratory illness	Total RSV cases: 170 RSV-A cases: 95 RSV-B cases: 62 Un-typed cases: 13	RSV-A was associated with significantly greater severity of illness, compared with RSV-B (*p* < 0.01) Mechanical ventilation requirement (*p* = 0.01), carbon dioxide tension > 45 mmHg (*p* = 0.04) were higher in RSV-A infection compared with RSV-B.
Hall et al. [40]	15 years (1975–1990)	Rochester, New York, United States	Hospitalized and ambulatory children aged 0–2 years with RSV infection	Total RSV cases: 1209 RSV-A cases: 858 RSV-B cases: 351	RSV-A was associated with higher risk of admission to the intensive care unit (*p* < 0.01).
Papadopoulos et al. [41]	1 winter season (from October 1999 to September 2000)	Athens, Greece	Hospitalized infants with RSV-positive bronchiolitis	Total RSV cases: 81 RSV-A cases: 23 cases RSV-B cases: 25 cases Un-typed: 33 cases	Disease severity index (assessed based on heart rate, respiratory rate, wheezing, cyanosis, difficulty of feeding and oxygen saturation) was higher in RSV-A bronchiolitis than in RSV-B induced one (*p* = 0.031). No statistically significant difference arose concerning the length of hospital stay and the need of intensive care.
Jafri et al. [42]	2 winter seasons (2006–2008)	United Sates (27 sites across 20 states)	Infants presenting to the Emergency Department with symptoms of lower respiratory tract infection or apnoea	Total RSV cases: 1299 RSV-A cases: 853 RSV-B cases: 453	Patients with RSV-A positive bronchiolitis had a higher rates of hospitalization compared with those positive for RSV-B virus (*p* < 0.001).
Laham et al. [13]	3 winter seasons (2007–2010)	United States (16 sites across 12 states)	Children aged 0–2 years hospitalized with bronchiolitis	Total RSV cases: 1589 RSV-A cases: 925 RSV-B cases: 649 RSV-A and RSV-B co-infected cases: 15	RSV-A positive bronchiolitis had a higher risk of intensive care treatment (defined as admission to the intensive care unit and/or the use of mechanical ventilation) compared with those having RSV-B (*p* = 0.048). On the contrary, no differences arose among children with co-infecting viruses
Hornsleth et al. [43]	3 winter seasons (1993–1995)	Copenhagen, Denmark	Children aged 0–2 years hospitalized with RSV-positive lower respiratory tract infection	Total RSV cases: 105 RSV-A cases: 31 RSV-B cases: 54 Un-typed cases: 20	Infants aged 0–5 months infected with RSV-B virus had a higher length of hospital stay than those infected with RSV-B virus (*p* = 0.039). Children aged 0–2 years with RSV-B infections had more severe disease than those with RSV-A infections, as assessed by respiratory rate (*p* = 0.013) and the presence of an infiltrate on a chest radiograph (*p* = 0.039).
Tran et al. [44]	1 year (from April 2010 to May 2011)	Ho Chi Minh City, Vietnam	Children aged 0–15 years admitted to hospital for an acute respiratory infection with an onset of illness less than 7 days	Total RSV cases: 257 RSV-A cases: 235 RSV-B cases: 13 RSV-A and RSV-B co-infected cases: 9	Children infected with RSV-A virus had a higher clinical severity score than those infected with group B (*p* = 0.049)
Fodha et al. [18]	1 year (2005)	Central coast of Tunisia	Previously healthy infants hospitalized with RSV bronchiolitis	Total RSV cases: 81 RSV-A cases: 9 RSV-B cases: 60 Un-typed cases: 12	Disease severity correlated with chronologic age < 28 days and nasopharyngeal RSV viral load (*p* = 0.024), but did not correlate with RSV subgroup.
McIntosh et al. [19]	3 years	Sydney, Australia	Children aged 0–2 years hospitalized with RSV-positive bronchiolitis	Total RSV cases: 444 RSV-A cases: 337 RSV-B cases: 107	No difference in severity between RSV-A and RSV-B infection.
Liu et al. [45]	3 years (2013–2015)	Guangzhou, China	Children aged 0–14 years hospitalized with RSV respiratory illness	Total RSV-cases: 729 RSV-A cases: 373 RSV-B cases: 356	Bronchiolitis (*p* < 0.001), dyspnea (*p* = 0.048), coryza (*p* < 0.001), vomiting (*p* = 0.001), poor appetite (*p* < 0.001), and diarrhea (*p* = 0.005) were more frequent in the RSV-A-positive patients than in the RSV-B-positive patients. Systemic influenza-like symptoms: chills (*p* = 0.01), headache (*p* = 0.006), myalgia (*p* = 0.002), debility (*p* = 0.006), and rash (*p* < 0.001) were more frequent in the RSV-B-positive patients than in the RSV-A-positive patients.

**Table 2:** Principal studies evaluating the correlation between RSV genotypes and severity of illness in children.

Study	Period of Study	Country	Study Population	Number of Cases	Results (*p* Value)
Martinello et al. [16]	2 winter seasons (1998–2000)	New Haven, Connecticut	Children aged 0–2 years without predisposing comorbidities presenting to the Emergency Room with ARI	Total RSV cases: 107 RSV-A cases: 64 Genotypes: - GA2: 29 cases; - GA3: 7 cases; - GA4: 24 cases. RSV-B cases: 43	No differences between RSV-A and B groups regarding the severity of illness (*p =* 0.590). GA3 genotype was associated with significantly greater severity of illness, compared with genotype GA2 (*p* = 0.018) and subgroup B (*p* = 0.032).
Yoshihara et al. [15]	3 years (from January 2010 to December 2012)	Nha Trang City, Central Vietnam	Children with RSV-related ARI	Total RSV cases: 362 RSV-A cases: 269 Genotypes: - ON1: 123 cases; - NA1: 138 cases; - Untyped: 8 cases. - RSV-B: 93 cases. Genotypes: - BA9: 57 cases; - BA10: 12 cases; - BA-C: 12 cases; - Untyped: 12 cases.	Shorter mean period from disease onset to hospital admission was seen in ON1 ARI cases (*p* < 0.001); Mean length of hospital stay (in days) between ON1 and NA1 ARI cases did not differ significantly (*p* = 0.329). Risk of wheezing was 2.21 (95% CI: 1.72–2.86) times, LRTI was 2.26 (95% CI: 1.37–3.72) times, and chest X-ray abnormality was 2.14 (95% CI: 1.13–4.04) times greater among ON1 ARI cases compared to NA1 ARI cases.
Esposito et al. [14]	5 winter seasons (2009–2014)	Milan, Italy	Children aged 0–2 years attending the Emergency Room because of influenza-like illness	Total RSV cases: 165 RSV-A cases: 131 Genotypes: - NA1: 62 cases; - ON1-A: 29 cases; - ON1-A1: 16 cases; - ON1-B: 24 cases. RSV-B cases: 34. Genotypes: - BA-9: 26 cases; - BA-10: 8 cases	No differences arose between RSV-A and B groups. Children infected by genotype A-NA1 more frequently had lower respiratory tract infections (*p* < 0.0001) and required hospitalisation (*p* = 0.007) than those infected by genotype A-ON1.
Tabatabai et al. [36]	1 winter season (2012–2013)	Heidelberg, Germany	Children aged 0–2 years hospitalized with upper or lower acute respiratory infection (ARI)	Total RSV cases: 134 RSV-A cases: 110 Genotypes: - ON1: 73 cases; - NA1: 23 cases; - GA5: 1 case; - Untyped: 13 cases. RSV-B cases: 24. Genotypes: - BA-IX: 10 cases; - BA-X 5 cases; - Untyped: 9 cases.	No difference arose between RSV groups and genotypes regarding symptoms prior to hospitalization in days (*p* = 0.98), hospital stay in days (*p* = 0.68) or need for intensive care (*p* = 0.49).
Panayiotou et al. [49]	3 winter seasons (2010–2013)	Cyprus	Children aged < 12 years hospitalized for ARI	Total RSV cases: 124 RSV-A cases: 83 Genotypes: - GA2: 28 cases; - ON1: 55 cases. RSV-B cases: 32 Genotype: - BA: 32 cases; Untyped: 9 cases	Genotype ON-1 was associated with less severe disease than GA2 and BA genotypes (*p* = 0.49).
Luchsinger et al. [48]	2 winter seasons (2010–2011)	Santiago, Chile	Previously healthy term infants, younger than 6 months of age, with a normal weight at birth, having their first acquired-community lower ARI	Total RSV cases: 74 RSV-A cases: 19 Genotype: - NA1: 19 cases RSV-B cases: 14. Genotypes: - B7: 13 cases; - B9: 1 cases Untyped: 41 cases	NA1 strains were more frequent in hospitalized infants (*p* < 0.001) and were associated with more severe course of illness (*p* = 0.01).
